# Supplementary material for: Fascin structural plasticity mediates flexible actin bundle construction
Source: bioRxiv. 2024 Feb 10:2024.01.03.574123. Originally published 2024 Jan 4. Preprint. [Version 2] doi: 10.1101/2024.01.03.574123 (PMC10802278; doi:10.1101/2024.01.03.574123)
Supplement: Supplement 5 [file NIHPP2024.01.03.574123v2-supplement-5.pdf]

bioRxiv preprint doi: https://doi.org/10.1101/2024.01.03.574123; this version posted February 11, 2024. The copyright holder for this preprint (which was not certified by peer review) is the author/funder, who has granted bioRxiv a license to display the preprint in perpetuity. It is made available under aCC-BY 4.0 International license.

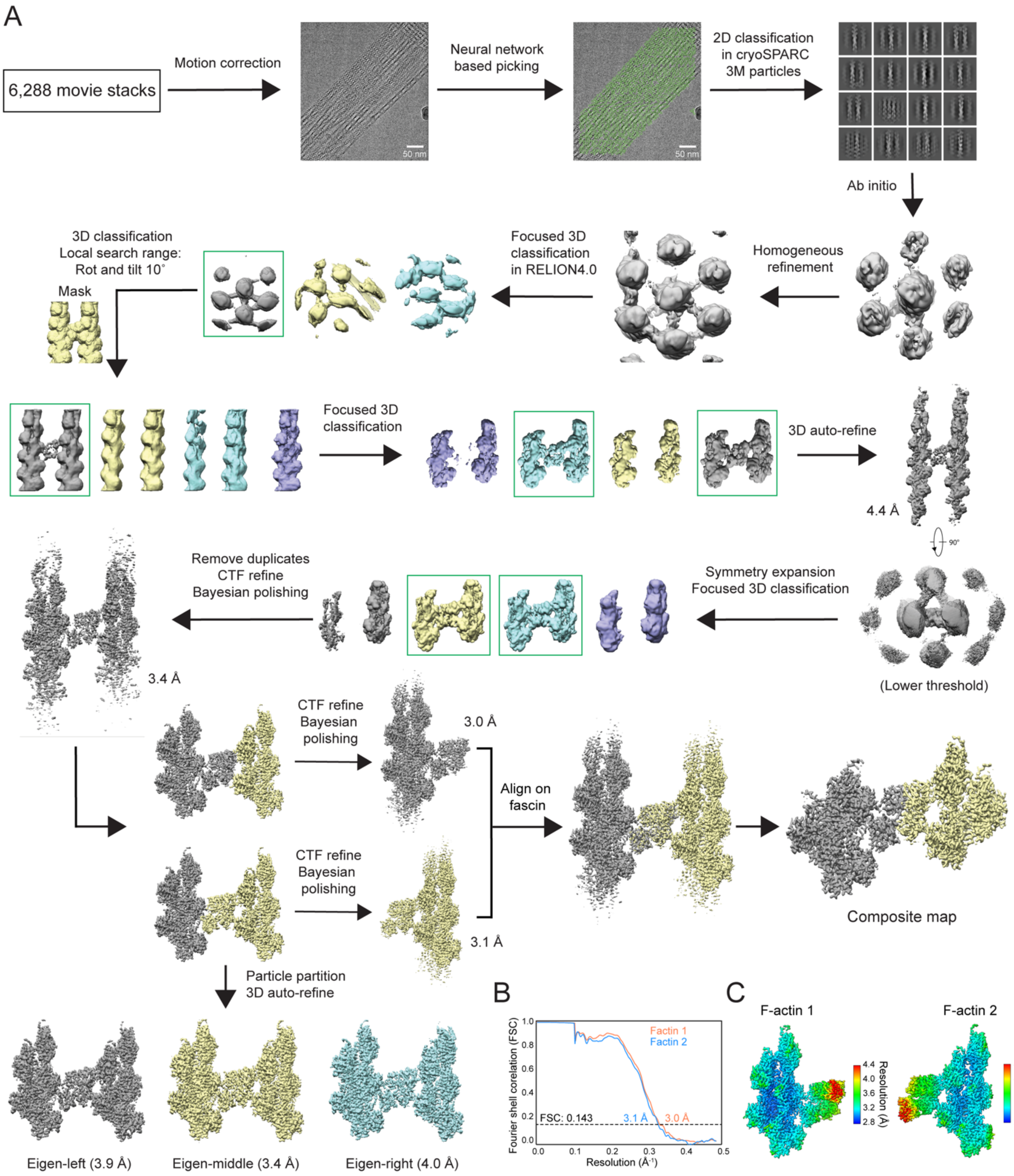

Figure S1. Cryo-EM data processing workflow

(A) Scheme of high resolution fascin crossbridge reconstruction data processing, including multibody refinement. (B) Fourier shell correlation (FSC) curves. (C) Local resolution assessment.

bioRxiv preprint doi: https://doi.org/10.1101/2024.01.03.574123; this version posted February 11, 2024. The copyright holder for this preprint (which was not certified by peer review) is the author/funder, who has granted bioRxiv a license to display the preprint in perpetuity. It is made available under aCC-BY 4.0 International license.

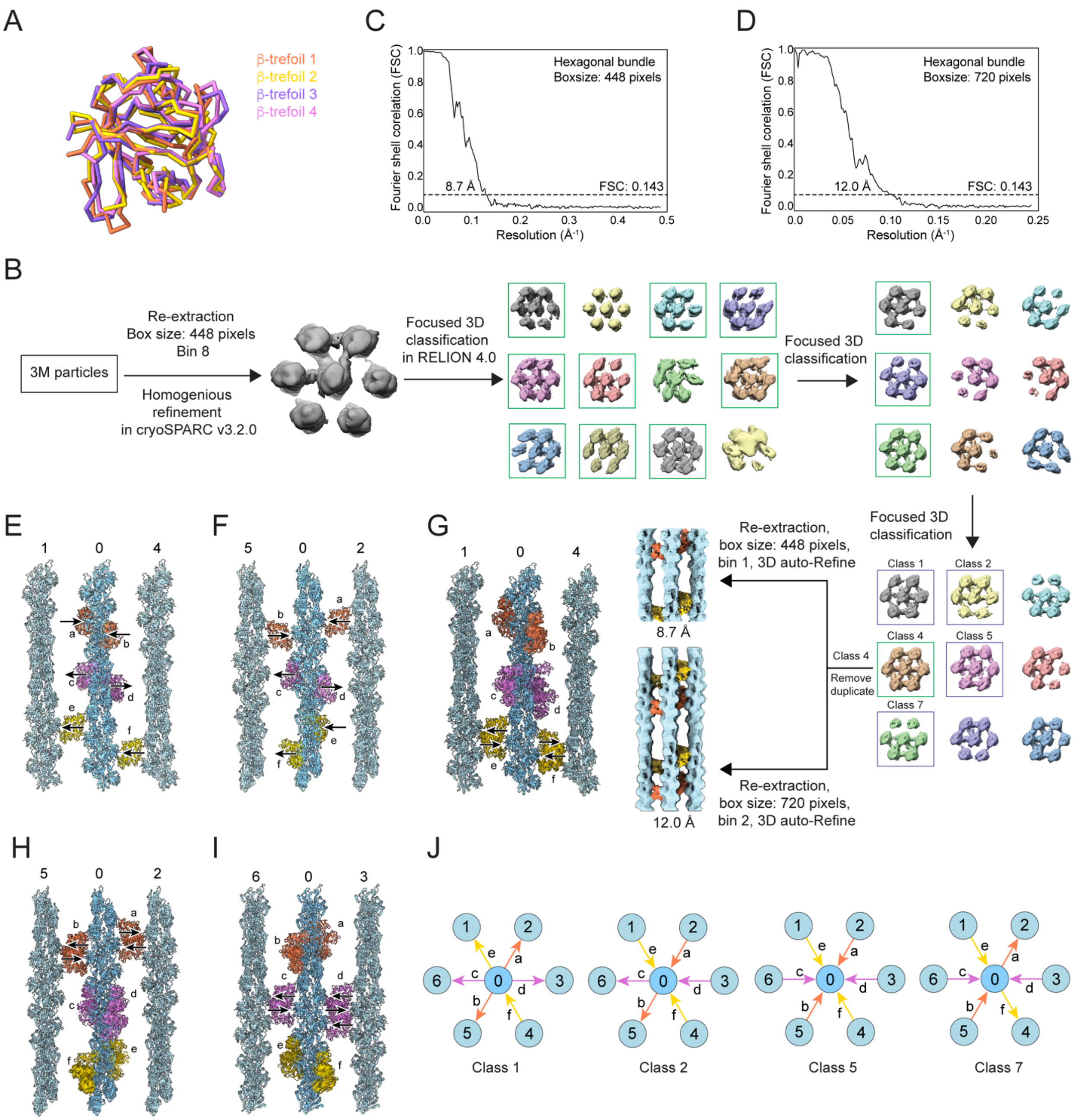

Figure S2. Analyses of fascin conformation and bundle element architecture
(A) Superposition of fascin's four individual β-trefoil domains extracted from the consensus atomic model (Figure 1F). (B) Cryo-EM data processing workflow for reconstructing a hexagonal bundle element. (C-D) FSC curves for the bundle element reconstructed with two different box sizes. (E-F) Side views of actin planes 1-0-4 (E) and 5-0-2 (F) from the 8.7 Å reconstruction docking model. (G-I) Side view of each actin plane of models from 5 bundle element classes aligned on the central filament. (J) Distributions of poses of the 6 central-filament associated fascins from the four additional bundle element classes.

bioRxiv preprint doi: https://doi.org/10.1101/2024.01.03.574123; this version posted February 11, 2024. The copyright holder for this preprint (which was not certified by peer review) is the author/funder, who has granted bioRxiv a license to display the preprint in perpetuity. It is made available under aCC-BY 4.0 International license.

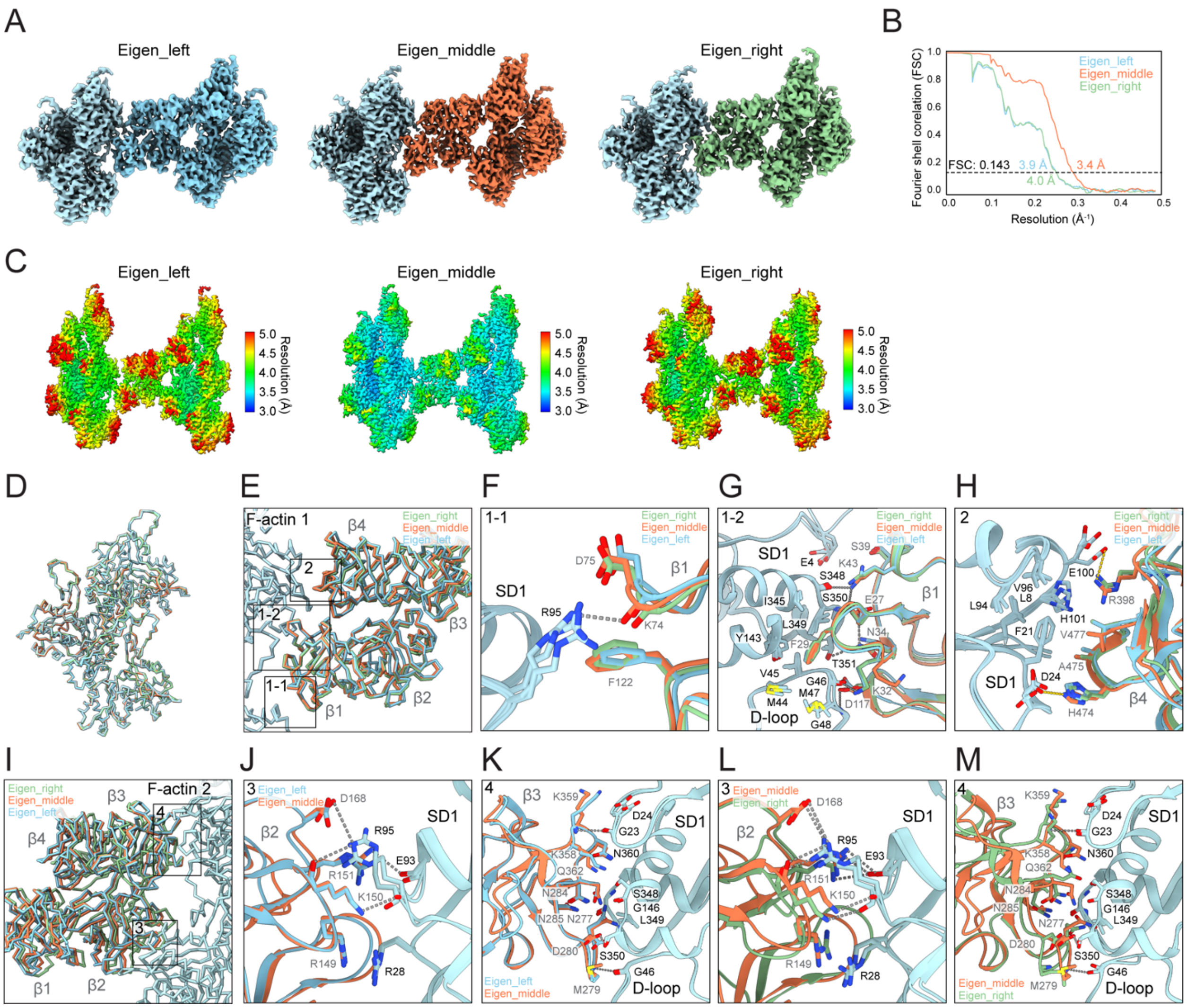

Figure S3. Analyses of multi-body derived reconstructions

(A) Cryo-EM density maps of eigen_left, eigen_middle and eigen_right reconstructions. (B-C) FSC curves (B) and local resolution assessment (C) of the three reconstructions. (D) Superposition of all six F-actin models from the three reconstructions. All F-actin 1 models are colored light blue, while F-actin 2 models are colored as in Figure 5B. (E) Comparison of the fascin-F-actin 1 interface across the three snapshots, superimposed on F-actin 1. (F-H) Detail views of contacts indicated in E. (I) Comparison of the fascin-F-actin 2 interface across the three snapshots, superimposed on F-actin 2. (J-M) Detail views of contacts indicated in I.

bioRxiv preprint doi: https://doi.org/10.1101/2024.01.03.574123; this version posted February 11, 2024. The copyright holder for this preprint (which was not certified by peer review) is the author/funder, who has granted bioRxiv a license to display the preprint in perpetuity. It is made available under aCC-BY 4.0 International license.

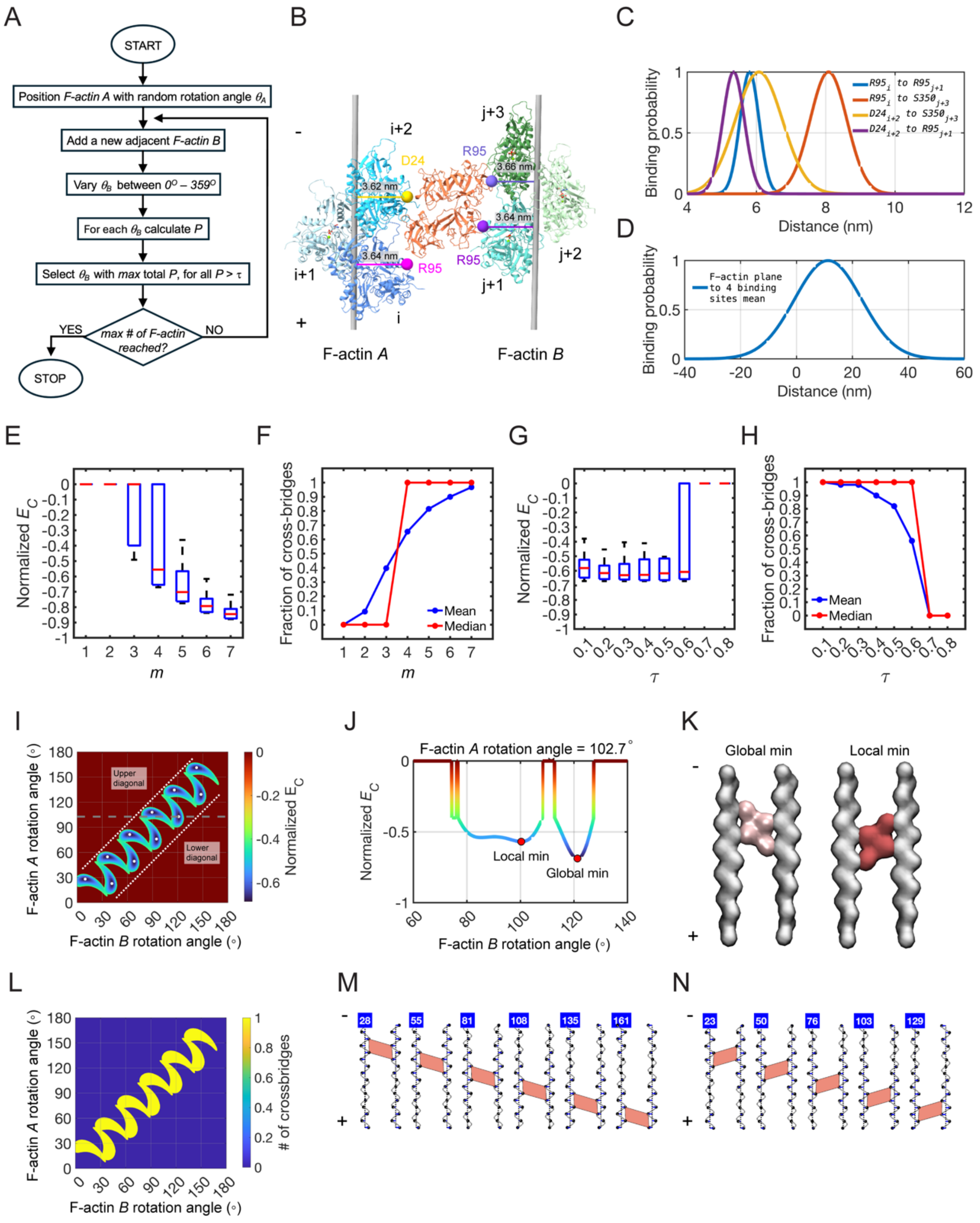

Figure S4. Computational model parametrization and analyses
(A) Flowchart of the computational model. Filaments are added iteratively, and the crosslinking probabilities for all angular rotations, θ_B, of the new filament *B* are evaluated. The angular rotation corresponding to the maximum probability of crosslinking is selected, as long as this probability is greater than the minimum cross-linking threshold, τ. (B) Atomic model of fascin crosslinked F-actin in ribbon representation. Filament *A* is depicted in shades of blue, while filament *B* is depicted in shades of green. Interfacial residues used as fiducials to calculate characteristic distances are indicated with spheres of varying colors. Grey cylinders represent F-actin axes. (C) Fascin binding probabilities as a function of

bioRxiv preprint doi: https://doi.org/10.1101/2024.01.03.574123; this version posted February 11, 2024. The copyright holder for this preprint (which was not certified by peer review) is the author/funder, who has granted bioRxiv a license to display the preprint in perpetuity. It is made available under aCC-BY 4.0 International license.

distances between fiducial residues. (D) Fascin binding probability as a function of the separation between the geometric center of the four fiducial residues and the plane which spans both filament axes. (E) Distribution of normalized crossbridge energies for different values of the standard deviation multiplier, *m*, between 1 and 7. N = 2,800 independent runs of filament pairs. Filament *A* was initialized with random absolute rotation angles, while τ was varied systematically between 0.1 and 0.8 in increments of 0.1. Red line indicates median; lower and upper bounds of box are 25th and 75th percentile, respectively. (F) Reanalysis of simulation results from E, plotting the fraction of crossbridges. (G) Boxplot of the distribution of normalized crossbridge energies for different values of τ using $m = 4$. N = 400 independent runs of filament pairs. F-actin *A* was initialized with random absolute rotation angles. Red line indicates median; lower and upper bounds of box are 25th and 75th percentile, respectively. (H) Reanalysis of simulation results from G, plotting the fraction of crossbridges. (I) Heatmap of the normalized cross-bridge energies for a filament pair as a function of absolute rotation angles. White dots show energy minima. (J) Plot of normalized crossbridge energy for F-actin *A* rotation angle = 102.7^{o} and varying the rotation angle of F-actin *B* between 60° and 140°, with increments of 0.1^{o}, corresponding to gray dashed line in I. (K) Surface representation of a fascin-crosslinked filament pair for the global and local minimum indicated in J. F-actins are represented in grey. For the global minimum, fascin is in pink and corresponds to $P = 0.69$. For the local minimum, fascin is in red, corresponding to $P = 0.57$. (L) Heatmap of the number of crossbridges for filament pairs with different rotational shifts. Each filament has 14 protomers. (M) Representative model renderings of the minima in the upper diagonal in I, showing fascin crossbridges in the "down" pose. (N) Representative model renderings of the minima in the lower diagonal in panel I, showing fascin crossbridges in the "up" pose. Blue boxes indicate filament *A* rotation angles.

bioRxiv preprint doi: https://doi.org/10.1101/2024.01.03.574123; this version posted February 11, 2024. The copyright holder for this preprint (which was not certified by peer review) is the author/funder, who has granted bioRxiv a license to display the preprint in perpetuity. It is made available under aCC-BY 4.0 International license.

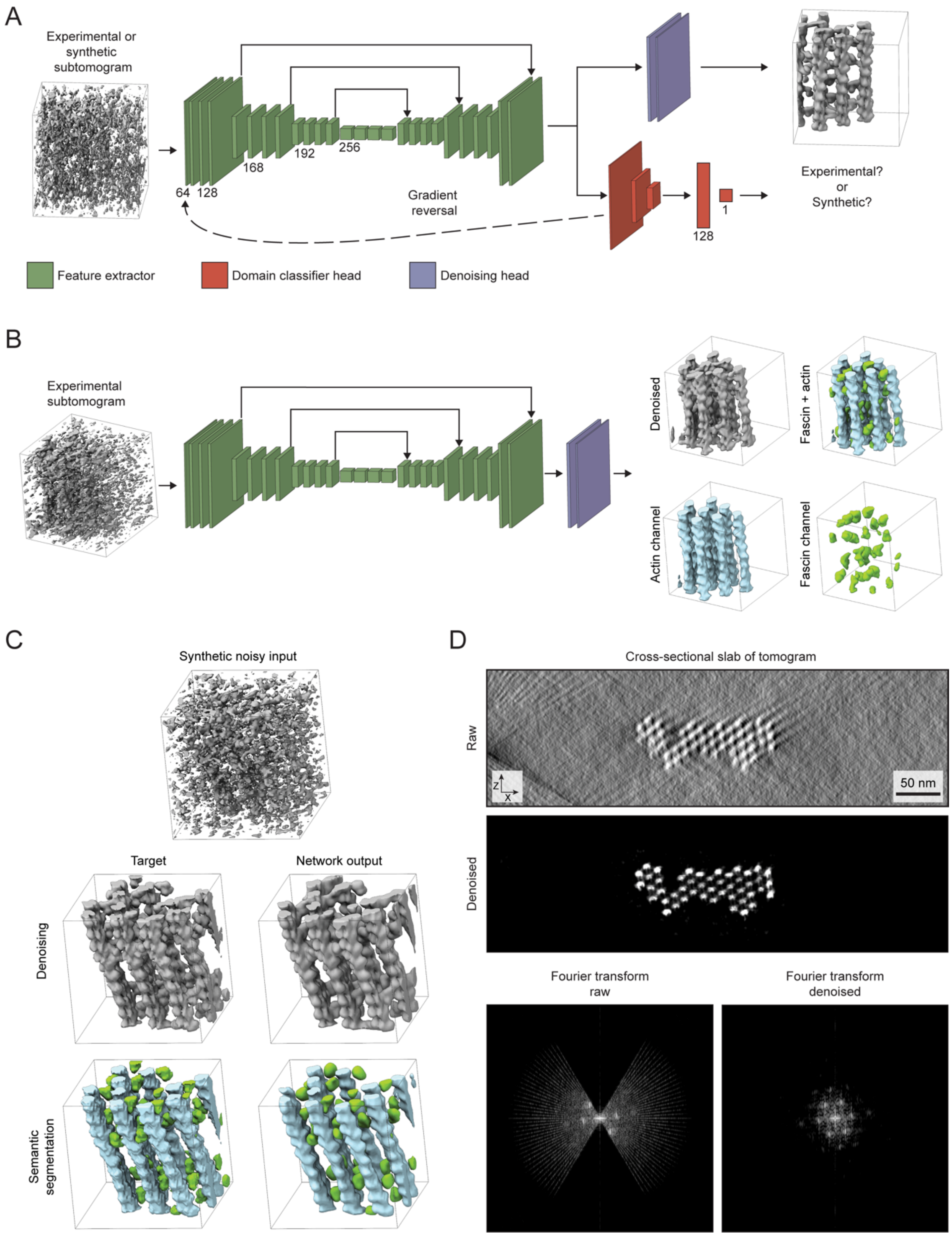

Figure S5. Neural network architecture and performance
(A) Neural network architecture used for pretraining on synthetic data and training on both synthetic and experimental subtomograms. (B) Neural network architecture and performance used for inference on experimental subtomogram. (C) Representative denoising and semantic segmentation performance on synthetic, noisy subtomogram. (D) Neural network denoising performance on cross-sectional slab of experimental tomogram.

bioRxiv preprint doi: https://doi.org/10.1101/2024.01.03.574123; this version posted February 11, 2024. The copyright holder for this preprint (which was not certified by peer review) is the author/funder, who has granted bioRxiv a license to display the preprint in perpetuity. It is made available under aCC-BY 4.0 International license.

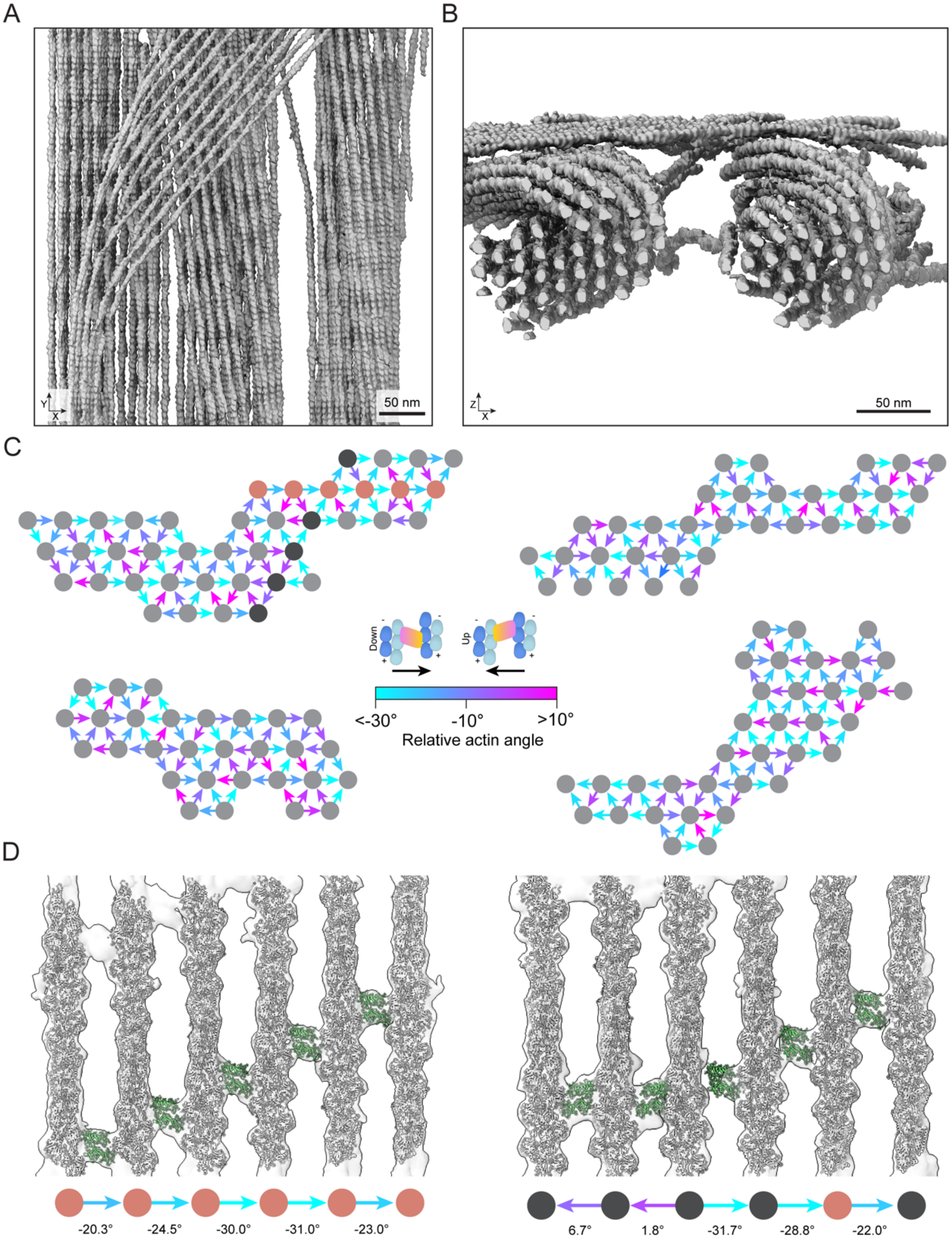

Figure S6. Additional structural analysis of fascin crosslinked bundles
(A) Top view of semantically segmented F-actin highlighting interconnected bundles. (B) End-on view of bundles in A, highlighting supertwist along their respective longitudinal axes. (C) End-on view schematics of filament rotational phase shifts and fascin poses of four additional bundles, analyzed as in Figure 6B. Dark grey and gold filaments correspond to actin planes displayed in D. (D) Side views of rigid-body docking models of two additional actin planes. Filament rotational phase offsets and fascin poses are indicated.

bioRxiv preprint doi: https://doi.org/10.1101/2024.01.03.574123; this version posted February 11, 2024. The copyright holder for this preprint (which was not certified by peer review) is the author/funder, who has granted bioRxiv a license to display the preprint in perpetuity. It is made available under aCC-BY 4.0 International license.

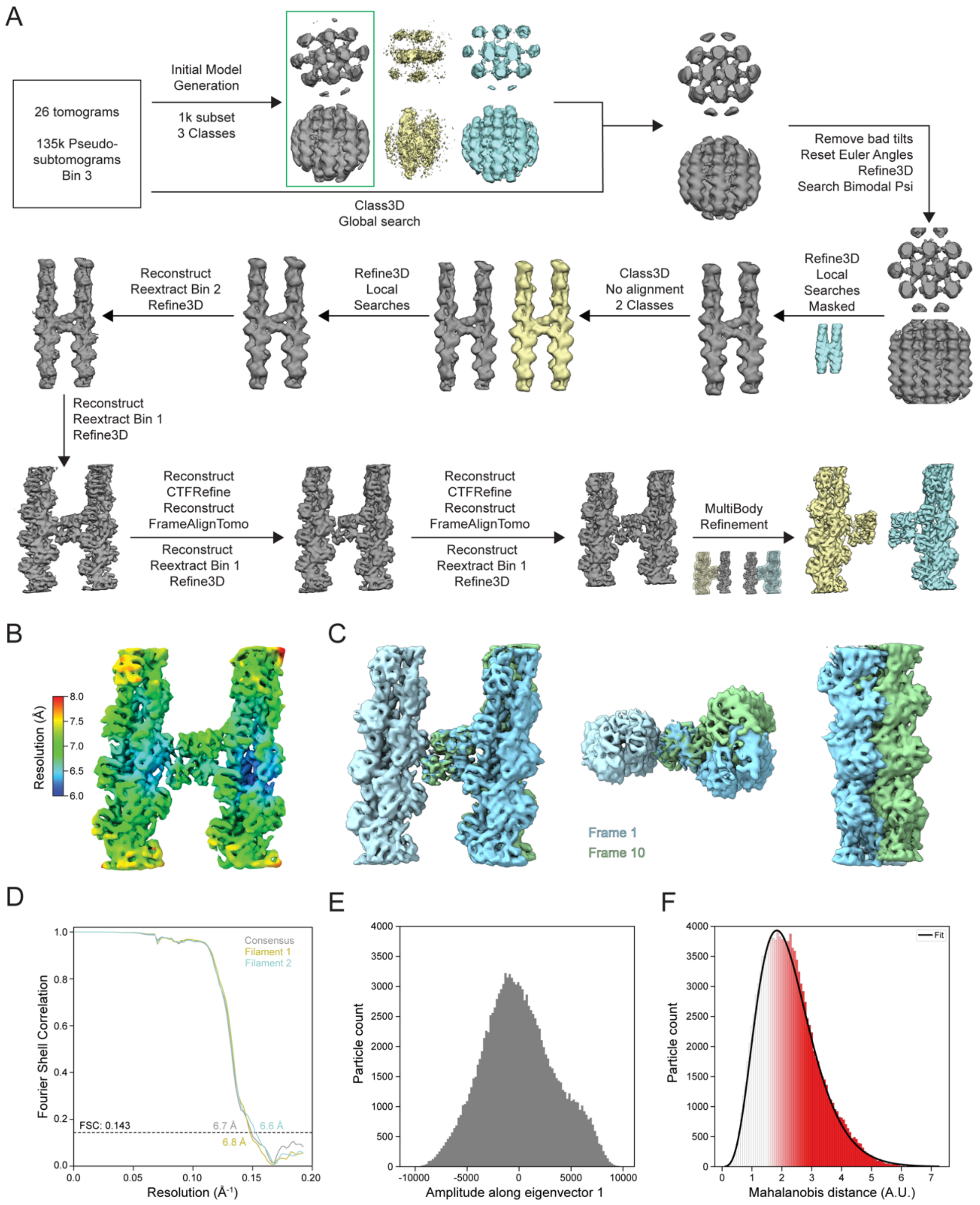

Figure S7. Subtomogram averaging workflow and analyses

(A) Subtomogram averaging data processing workflow. (B) Local resolution map of subtomogram average. (C) Extreme frames (1 representing 5th percentile, and 10 representing 95th percentile) of interpolation along the first principal component of multibody refinement. Similar inter-filament rotation is present as observed in single particle analysis. (D) FSC curves of the consensus (gray) and multi-body refinement reconstructions (yellow, blue). (E) Distribution of amplitudes along eigenvector 1 of all subtomograms. (F) Distribution of Mahalanobis distances of all subtomograms (n = 129,948). Fit represents gamma distribution ($R^2 = 0.9951$).

bioRxiv preprint doi: https://doi.org/10.1101/2024.01.03.574123; this version posted February 11, 2024. The copyright holder for this preprint (which was not certified by peer review) is the author/funder, who has granted bioRxiv a license to display the preprint in perpetuity. It is made available under aCC-BY 4.0 International license.

Table S1. Cryo-EM data collection, refinement, and validation statistics

**Data collection**								
Microscope	Titan Krios							
Voltage (kV)	300							
Detector	K2 Summit							
Magnification	29,000							
Electron exposure (e^- / Å^2)	61.26							
Exposure rate (e^- / pixel / s)	6.5							
Calibrated pixel size (Å)	1.03							
Defocus range (µm)	–0.8 to –2.2							
Symmetry imposed	C1							
**Data processing**	Fascin crosslinked F-actin						Bundle element	
	Multibody: F-actin 1	Multibody: F-actin 2	Composite map	Eigen_left	Eigen_middle	Eigen_right	Box: 460 Å	Box: 740 Å
Initial particle images (no.)	3,056,360						3,056,360	3,056,360
Final particle images (no.)	113,800			17,207	79,824	16,769	8,477	8,053
Map resolution (Å)	3.0	3.1		3.9	3.4	4.0	8.7	12.0
FSC threshold	0.143							
**Refinement**								
Initial model (PDB ID)	7R8V, 3LLP	7R8V, 3LLP	7R8V, 3LLP	7R8V, 3LLP	7R8V, 3LLP	7R8V, 3LLP		
Model resolution (Å)	3.1	3.0	3.1	3.6	3.5	3.6		
FSC threshold	0.5	0.5	0.5	0.5	0.5	0.5		
Map sharpening B factor (Å^2)	-55.11	-48.84		-50.28	-62.33	-48.48	-108.59	-512.65
Model composition	3 actin protomers, 1 fascin	3 actin protomers, 1 fascin	6 actin protomers, 1 fascin	6 actin protomers, 1 fascin	6 actin protomers, 1 fascin	6 actin protomers, 1 fascin		
Non-hydrogen atoms	12,625	12,625	21,460	21,460	21,460	21,460		
Protein residues	1,604	1,604	2,723	2,723	2,723	2,723		
Ligands	3 Mg.ADP	3 Mg.ADP	6 Mg.ADP	6 Mg.ADP	6 Mg.ADP	6 Mg.ADP		
B factors (Å^2)								
Protein	23.94	27.87	43.13	99.43	78.72	112.12		
Ligand	8.28	18.9	39.23	90.45	65.04	96.87		
R.M.S. deviations								
Bond lengths (Å)	0.003	0.005	0.004	0.004	0.002	0.004		
Bond angles (°)	0.583	0.663	0.612	0.667	0.566	0.614		
**Validation**								
MolProbity score	1.55	1.73	1.55	1.94	1.63	2.07		
Clash score	7.37	8.65	8.07	16.31	8.24	18.66		
Poor rotamers (%)	0	0	0	0.04	0.07	0.09		
Ramachandran plot								
Favored (%)	97.23	96.16	97.44	96.58	96.92	95.62		
Allowed (%)	2.77	3.84	2.56	3.42	3.08	4.38		
Disallowed (%)	0.00	0.00	0.00	0.00	0.00	0.00		

bioRxiv preprint doi: https://doi.org/10.1101/2024.01.03.574123; this version posted February 11, 2024. The copyright holder for this preprint (which was not certified by peer review) is the author/funder, who has granted bioRxiv a license to display the preprint in perpetuity. It is made available under aCC-BY 4.0 International license.

Table S2. Cryo-ET data collection and subtomogram averaging

**Data collection**			
Microscope	Titan Krios		
Voltage (kV)	300		
Detector	K3		
Magnification	26,000		
Electron exposure (e^- / Å^2)	109.17		
Exposure rate (e^- / pixel / s)	30		
Calibrated pixel size (Å)	2.6		
Defocus (μm)	–4.0		
Symmetry Imposed	C1		
**Data Processing**	Multibody: F-actin 1	Multibody: F-actin 2	Composite map
Initial particle images (no.)	134,733		
Final particle images (no.)	129,948		
Map resolution (Å)	6.8	6.6	6.7
FSC threshold	0.143	0.143	0.143
Map sharpening B factor (Å^2)	-301.274	-280.458	-233.239

Table S3. Computational modeling probability function parameters

**Function name**	**Distance assessed**	**Mean (μ, nm)**	**Standard deviation (σ, nm)**
P1	$R95_i$ to $R95_{j+1}$	5.80	0.069
P2	$R95_i$ to $S350_{j+3}$	8.08	0.133
P3	$D24_{i+2}$ to $S350_{j+3}$	6.07	0.170
P4	$D24_{i+2}$ to $R95_{j+1}$	5.34	0.078
P5	Center of fiducial residues to plane spanning both filament axes	11.287	3.0

Supplementary Video Captions

Video S1: Morphs between prebound, inhibitor bound, and F-actin bound fascin conformations
Structures are superimposed on fascin β-trefoil 2.

Video S2: Overview of fascin crosslinked F-actin bundle element architecture

Video S3: Morph between eigen_left, eigen_middle, and eigen_right conformational snapshots
Structures are superimposed on F-actin 1.

Video S4: Overview of a tomogram showcasing denoising and semantic segmentation
