# Supplementary material for: Fascin structural plasticity mediates flexible actin bundle construction
Source: bioRxiv. 2024 Jan 4:2024.01.03.574123. Preprint. [Version 1] doi: 10.1101/2024.01.03.574123 (PMC10802278; doi:10.1101/2024.01.03.574123)
Supplement: Supplementary file 1 [file NIHPP2024.01.03.574123v1-supplement-4.pdf]

bioRxiv preprint doi: https://doi.org/10.1101/2024.01.03.574123; this version posted January 4, 2024. The copyright holder for this preprint (which was not certified by peer review) is the author/funder, who has granted bioRxiv a license to display the preprint in perpetuity. It is made available under aCC-BY 4.0 International license.

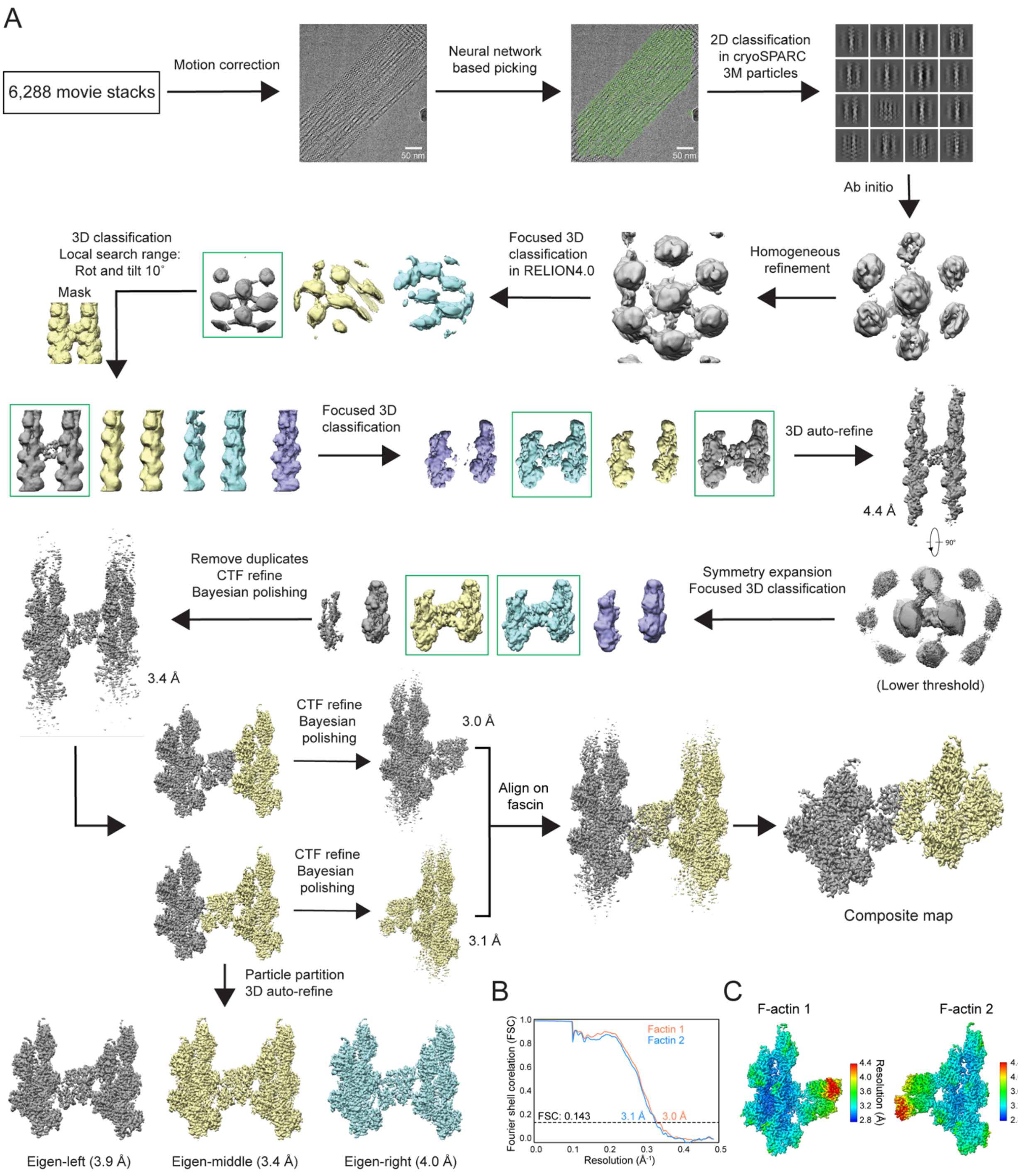

Figure S1. Cryo-EM data processing workflow
(**A**) Scheme of high resolution fascin crossbridge reconstruction data processing, including multibody refinement. (**B**) Fourier shell correlation (FSC) curves. (**C**) Local resolution assessment.

bioRxiv preprint doi: https://doi.org/10.1101/2024.01.03.574123; this version posted January 4, 2024. The copyright holder for this preprint (which was not certified by peer review) is the author/funder, who has granted bioRxiv a license to display the preprint in perpetuity. It is made available under aCC-BY 4.0 International license.

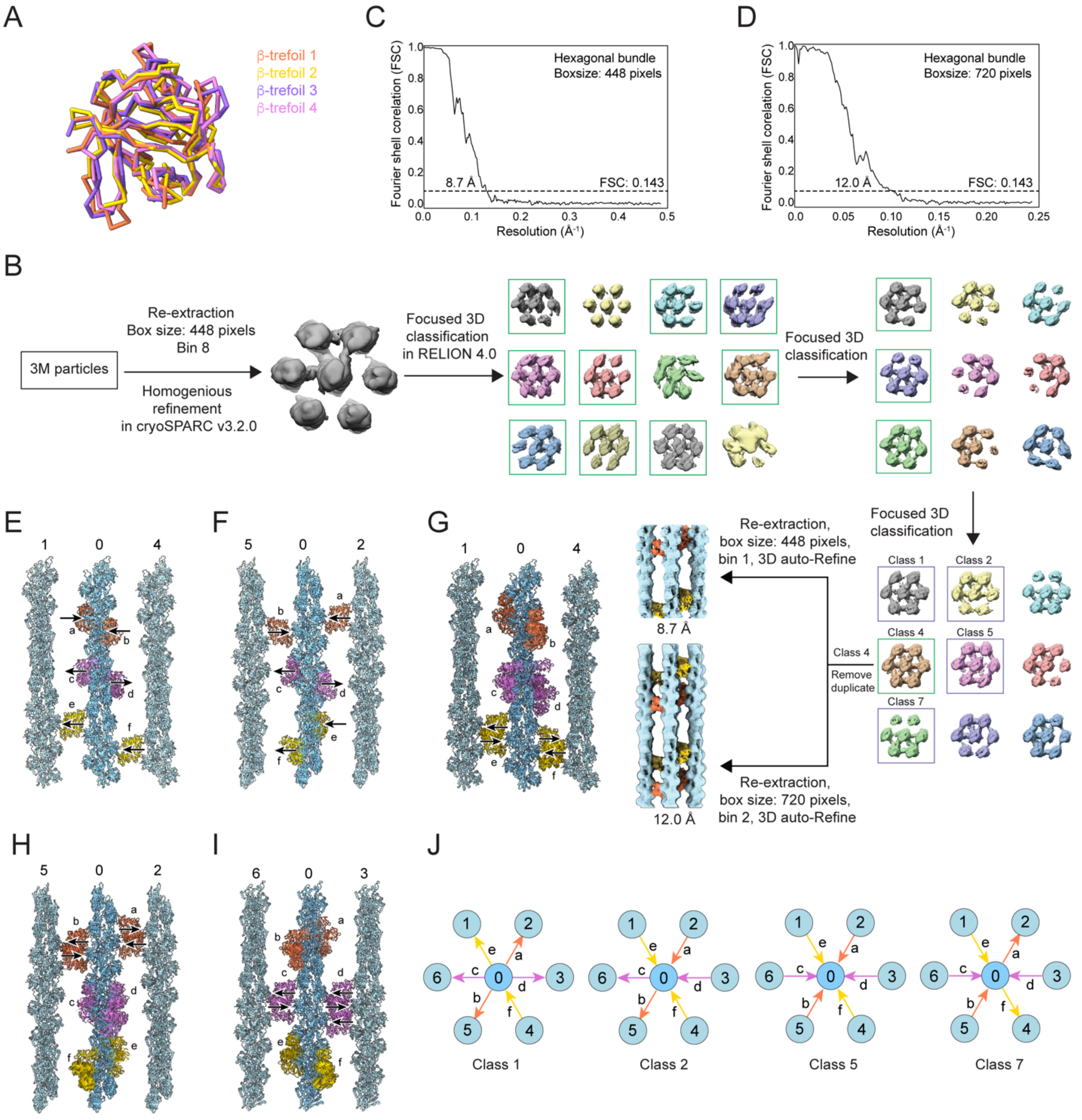

Figure S2. Analyses of fascin conformation and bundle element architecture
(**A**) Superposition of fascin's four individual β-trefoil domains extracted from the consensus atomic model (Figure 1F). (**B**) Cryo-EM data processing workflow for reconstructing a hexagonal bundle element. (**C** and **D**) FSC curves for the bundle element reconstructed with two different box sizes. (**E** and **F**) Side views of actin planes 1-0-4 (**E**) and 5-0-2 (**F**) from the 8.7Å reconstruction docking model. (**G-I**) Side view of each actin plane of models from 5 bundle element classes aligned on the central filament. (**J**) Distributions of poses of the 6 central-filament associated fascins from the four additional bundle element classes.

bioRxiv preprint doi: https://doi.org/10.1101/2024.01.03.574123; this version posted January 4, 2024. The copyright holder for this preprint (which was not certified by peer review) is the author/funder, who has granted bioRxiv a license to display the preprint in perpetuity. It is made available under aCC-BY 4.0 International license.

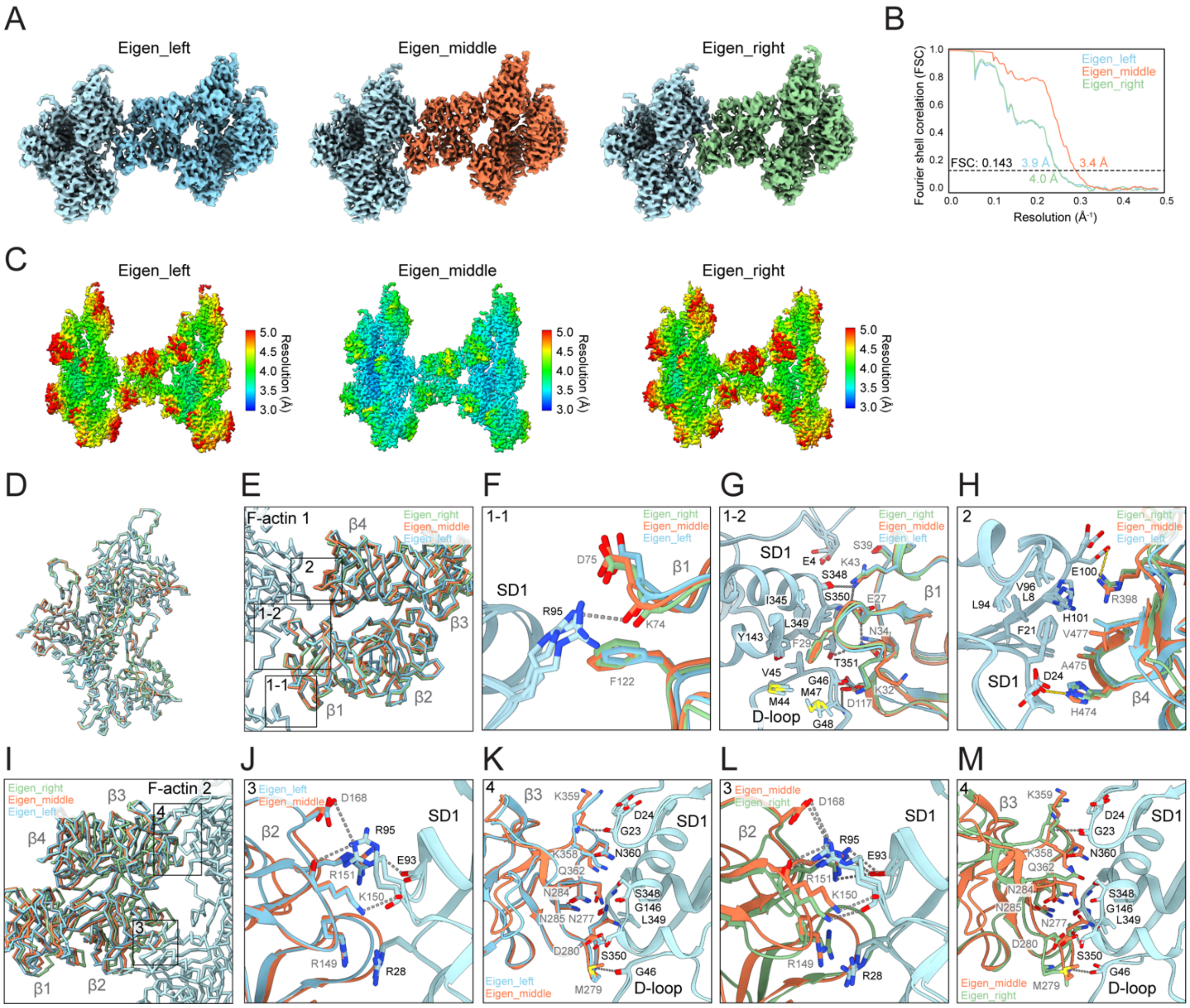

Figure S3. Analyses of multi-body derived reconstructions

(**A**) Cryo-EM density maps of eigen_left, eigen_middle and eigen_right reconstructions. (**B** and **C**) FSC curves (**B**) and local resolution assessment (**C**) of the three reconstructions. (**D**) Superposition of all six F-actin models from the three reconstructions. All F-actin 1 models are colored light blue, while F-actin 2 models are colored as in Figure 5B. (**E**) Comparison of the fascin-F-actin 1 interface across the three snapshots, superimposed on F-actin 1. (**F-H**) Detail views of contacts indicated in E. (**I**) Comparison of the fascin-F-actin 2 interface across the three snapshots, superimposed on F-actin 2. (**J-M**) Detail views of contacts indicated in I.

bioRxiv preprint doi: https://doi.org/10.1101/2024.01.03.574123; this version posted January 4, 2024. The copyright holder for this preprint (which was not certified by peer review) is the author/funder, who has granted bioRxiv a license to display the preprint in perpetuity. It is made available under aCC-BY 4.0 International license.

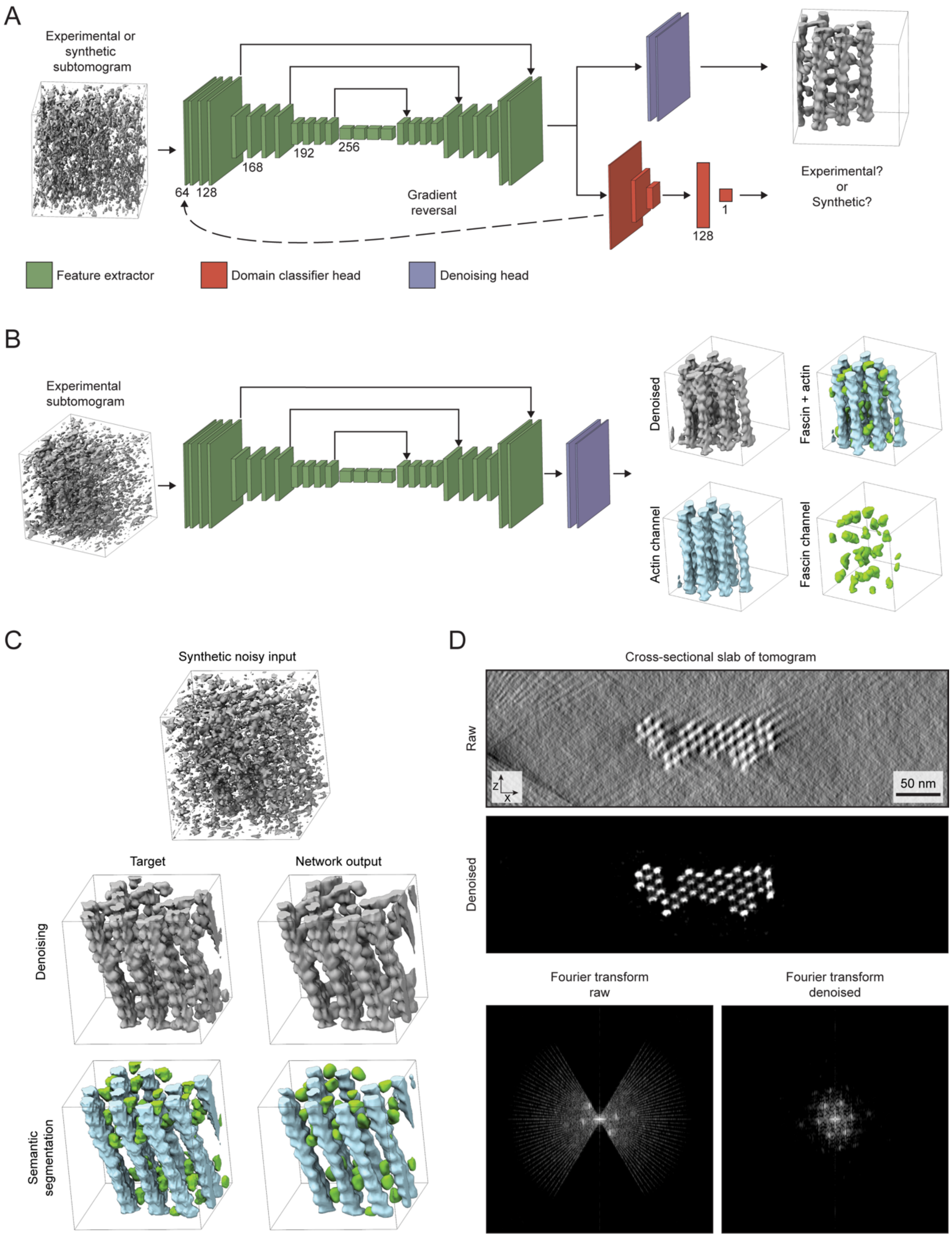

Figure S4. Neural network architecture and performance
(**A**) Neural network architecture used for pretraining on synthetic data and training on both synthetic and experimental subtomograms. (**B**) Neural network architecture and performance used for inference on experimental subtomogram. (**C**) Representative denoising and semantic segmentation performance on synthetic, noisy subtomogram. (**D**) Neural network denoising performance on cross-sectional slab of experimental tomogram.

bioRxiv preprint doi: https://doi.org/10.1101/2024.01.03.574123; this version posted January 4, 2024. The copyright holder for this preprint (which was not certified by peer review) is the author/funder, who has granted bioRxiv a license to display the preprint in perpetuity. It is made available under aCC-BY 4.0 International license.

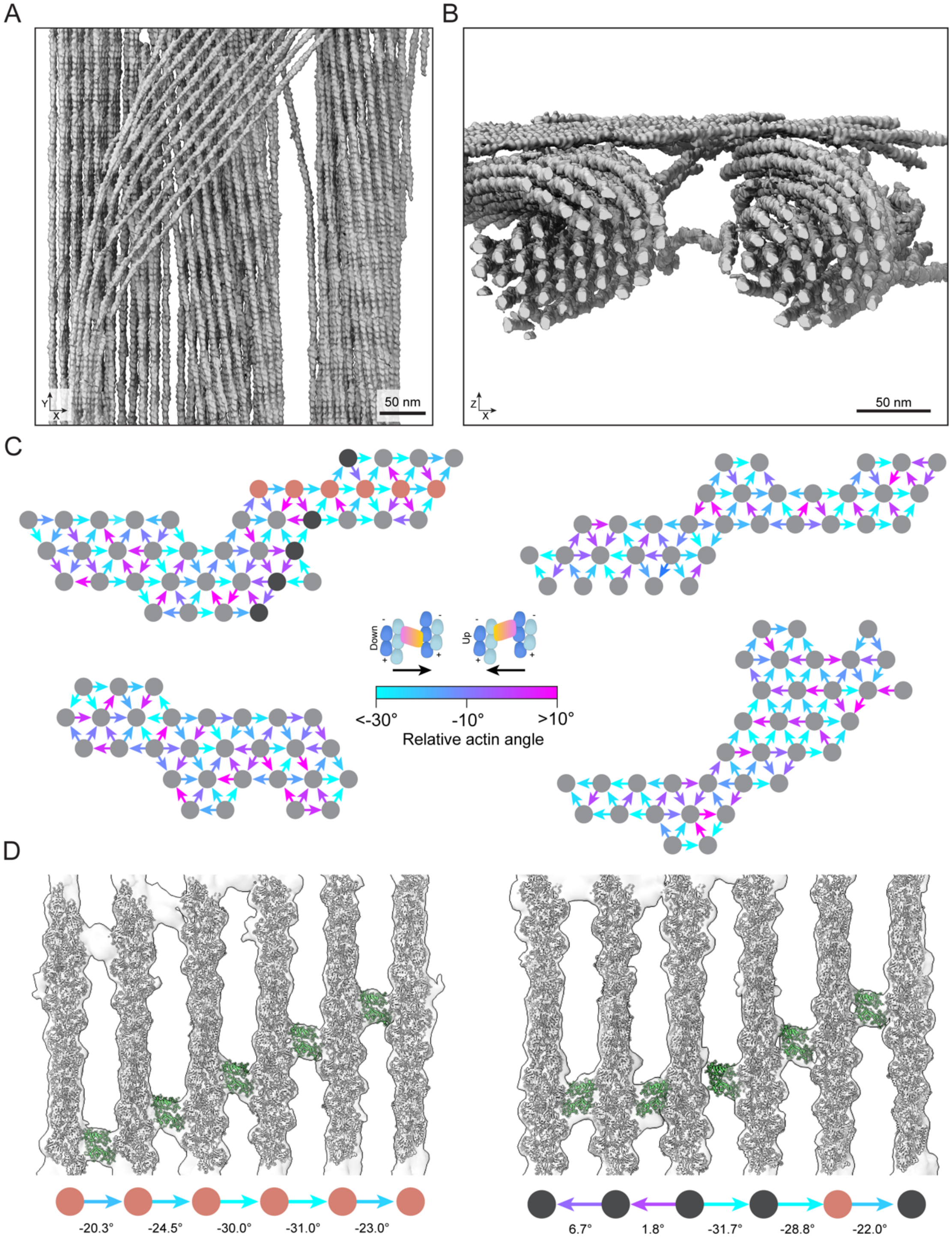

Figure S5. Additional structural analysis of fascin crosslinked bundles
(**A**) Top view of semantically segmented F-actin highlighting interconnected bundles. (**B**) End-on view of bundles in A, highlighting supertwist along their respective longitudinal axes. (**C**) End-on view schematics of filament rotational phase shifts and fascin poses of four additional bundles, analyzed as in Figure 6B. Dark grey and gold filaments correspond to actin planes displayed in D. (**D**) Side views of rigid-body docking models of two additional actin planes. Filament rotational phase offsets and fascin poses are indicated

bioRxiv preprint doi: https://doi.org/10.1101/2024.01.03.574123; this version posted January 4, 2024. The copyright holder for this preprint (which was not certified by peer review) is the author/funder, who has granted bioRxiv a license to display the preprint in perpetuity. It is made available under aCC-BY 4.0 International license.

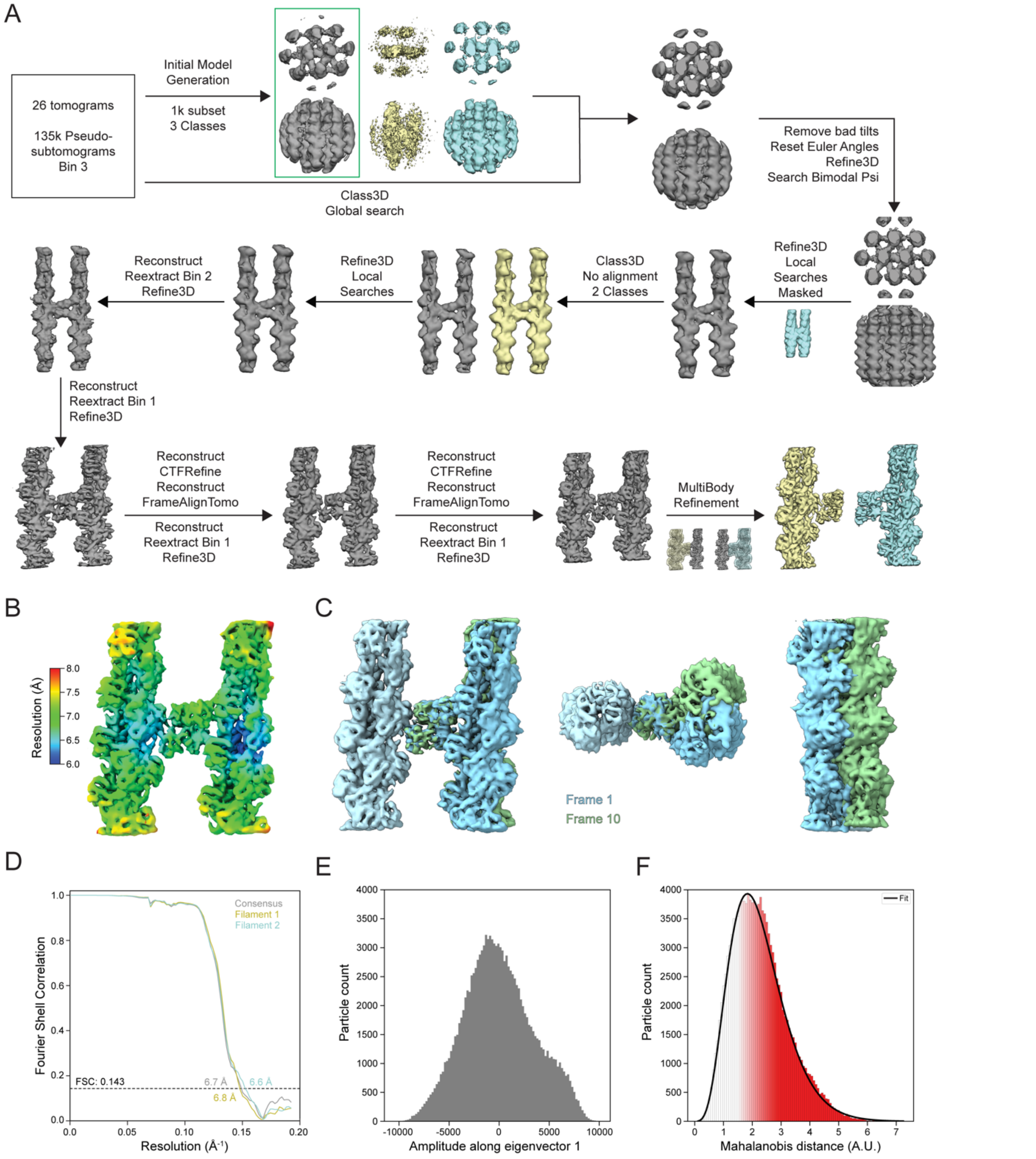

Figure S6. Subtomogram averaging workflow and analyses
(**A**) Subtomogram averaging data processing workflow. (**B**) Local resolution map of subtomogram average. (**C**) Extreme frames (1 representing 5th percentile, and 10 representing 95th percentile) of interpolation along the first principal component of multibody refinement. Similar inter-filament rotation is present as observed in single particle analysis. (**D**) FSC curves of the consensus (gray) and multi-body refinement reconstructions (yellow, blue). (**E**) Distribution of amplitudes along eigenvector 1 of all subtomograms. (**F**) Distribution of Mahalanobis distances of all subtomograms (n = 129,948). Fit represents gamma distribution (R^2 = 0.9951).

bioRxiv preprint doi: https://doi.org/10.1101/2024.01.03.574123; this version posted January 4, 2024. The copyright holder for this preprint (which was not certified by peer review) is the author/funder, who has granted bioRxiv a license to display the preprint in perpetuity. It is made available under aCC-BY 4.0 International license.

Table S1. Cryo-EM data collection, refinement, and validation statistics

**Data collection**								
Microscope	Titan Krios							
Voltage (kV)	300							
Detector	K2 Summit							
Magnification	29,000							
Electron exposure (e^{-} / Å^2)	61.26							
Exposure rate (e^{-} / pixel / s)	6.5							
Calibrated pixel size (Å)	1.03							
Defocus range (μm)	–0.8 to –2.2							
Symmetry imposed	C1							
**Data processing**	Fascin crosslinked F-actin						Bundle element	
	Multibody: F-actin 1	Multibody: F-actin 2	Composite map	Eigen_left	Eigen_middle	Eigen_right	Box: 460 Å	Box: 740 Å
Initial particle images (no.)	3,056,360						3,056,360	3,056,360
Final particle images (no.)	113,800			17,207	79,824	16,769	8,477	8,053
Map resolution (Å)	3.0	3.1		3.9	3.4	4.0	8.7	12.0
FSC threshold	0.143							
**Refinement**								
Initial model (PDB ID)	7R8V, 3LLP	7R8V, 3LLP	7R8V, 3LLP	7R8V, 3LLP	7R8V, 3LLP	7R8V, 3LLP		
Model resolution (Å)	3.1	3.0	3.1	3.6	3.5	3.6		
FSC threshold	0.5	0.5	0.5	0.5	0.5	0.5		
Map sharpening B factor (Å^2)	-55.11	-48.84		-50.28	-62.33	-48.48	-108.59	-512.65
Model composition	3 actin protomers, 1 fascin	3 actin protomers, 1 fascin	6 actin protomers, 1 fascin	6 actin protomers, 1 fascin	6 actin protomers, 1 fascin	6 actin protomers, 1 fascin		
Non-hydrogen atoms	12,625	12,625	21,460	21,460	21,460	21,460		
Protein residues	1,604	1,604	2,723	2,723	2,723	2,723		
Ligands	3 Mg.ADP	3 Mg.ADP	6 Mg.ADP	6 Mg.ADP	6 Mg.ADP	6 Mg.ADP		
B factors (Å^2)								
Protein	23.94	27.87	43.13	99.43	78.72	112.12		
Ligand	8.28	18.9	39.23	90.45	65.04	96.87		
R.M.S. deviations								
Bond lengths (Å)	0.003	0.005	0.004	0.004	0.002	0.004		
Bond angles (°)	0.583	0.663	0.612	0.667	0.566	0.614		
**Validation**								
MolProbity score	1.55	1.73	1.55	1.94	1.63	2.07		
Clash score	7.37	8.65	8.07	16.31	8.24	18.66		
Poor rotamers (%)	0	0	0	0.04	0.07	0.09		
Ramachandran plot								
Favored (%)	97.23	96.16	97.44	96.58	96.92	95.62		
Allowed (%)	2.77	3.84	2.56	3.42	3.08	4.38		
Disallowed (%)	0.00	0.00	0.00	0.00	0.00	0.00		

bioRxiv preprint doi: https://doi.org/10.1101/2024.01.03.574123; this version posted January 4, 2024. The copyright holder for this preprint (which was not certified by peer review) is the author/funder, who has granted bioRxiv a license to display the preprint in perpetuity. It is made available under aCC-BY 4.0 International license.

Table S2: Cryo-ET data collection and subtomogram averaging

**Data collection**			
Microscope	Titan Krios		
Voltage (kV)	300		
Detector	K3		
Magnification	26,000		
Electron exposure (e^{-} / Å^2)	109.17		
Exposure rate (e^{-} / pixel / s)	30		
Calibrated pixel size (Å)	2.6		
Defocus (μm)	–4.0		
Symmetry Imposed	C1		
**Data Processing**	Multibody: F-actin 1	Multibody: F-actin 2	Composite map
Initial particle images (no.)	134,733		
Final particle images (no.)	129,948		
Map resolution (Å)	6.8	6.6	6.7
FSC threshold	0.143	0.143	0.143
Map sharpening B factor (Å^2)	-301.274	-280.458	-233.239

Supplementary Video Captions

Video S1: Morphs between prebound, inhibitor bound, and F-actin bound fascin conformations
Structures are superimposed on fascin β-trefoil 2.

Video S2: Overview of fascin crosslinked F-actin bundle element architecture

Video S3: Morph between eigen_left, eigen_middle, and eigen_right conformational snapshots
Structures are superimposed on F-actin 1.
